# Supplementary material for: Oxidized-LDL Deteriorated the Renal Residual Function and Parenchyma in CKD Rat through Upregulating Epithelial Mesenchymal Transition and Extracellular Matrix-Mediated Tubulointerstitial Fibrosis—Pharmacomodulation of Rosuvastatin
Source: Antioxidants (Basel). 2022 Dec 15;11(12):2465. doi: 10.3390/antiox11122465 (PMC9774560; doi:10.3390/antiox11122465)
Supplement: Supplementary file 1 [file antioxidants-11-02465-s001.zip › antioxidants-2017021-supplementary.pdf]

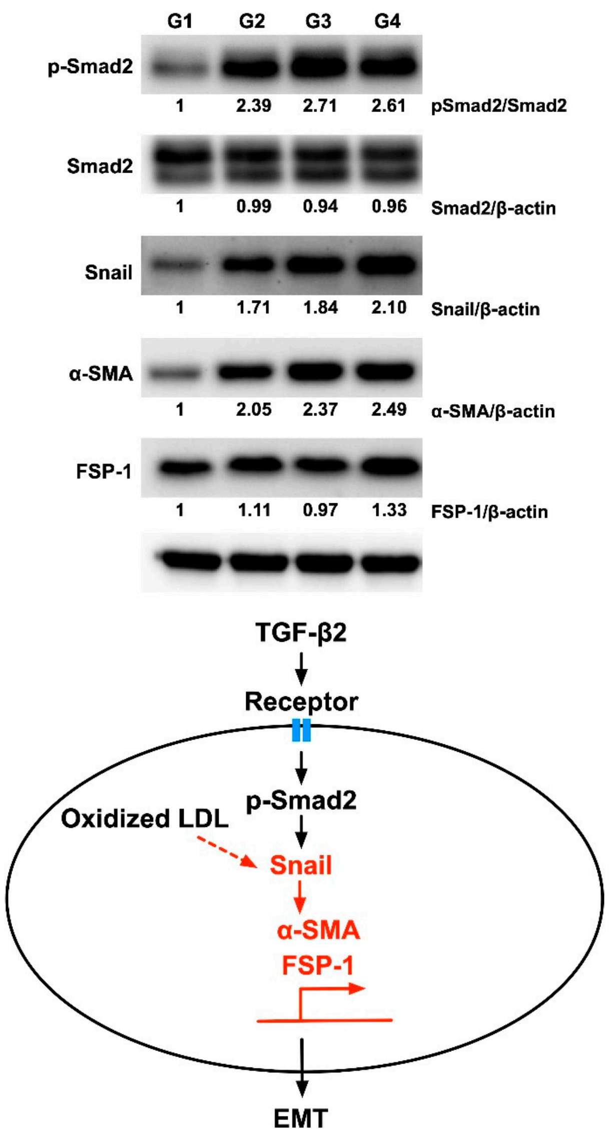


**Figure S1.** Preliminary results for providing a corrective direction of the study. Frist, prior to carried on the present study, we encouraged us to perform a preliminary data to support our hypothesis. Thus, we utilized the NRK-52E cells (i.e., rat proximal renal tubular cell line) and the cells were categorized into G1 (i.e., NRK-52E), G2 [NRK-52E + TGF-β (5ng/mL)], G3 [NRK-52E + TGF-β + oxidized LDL (5 ug/mL)] and G4 [NRK-52E + TGF-β+ oxidized LDL (20 ug/ml)], respectively. The result showed that the protein expressions of phosphorylated (p)-Smad2, snail, alpha smooth muscle actin (α-SMA), and fibroblast-specific protein 1 (Fsp1), four biomarkers of EMT, were notably progressively increased from G1 to G4. These findings provided essential information for us to schematically simplified illustration of the underling mechanism of EMT process undergoing oxidized LDL facilitated TGF-β stimulation.

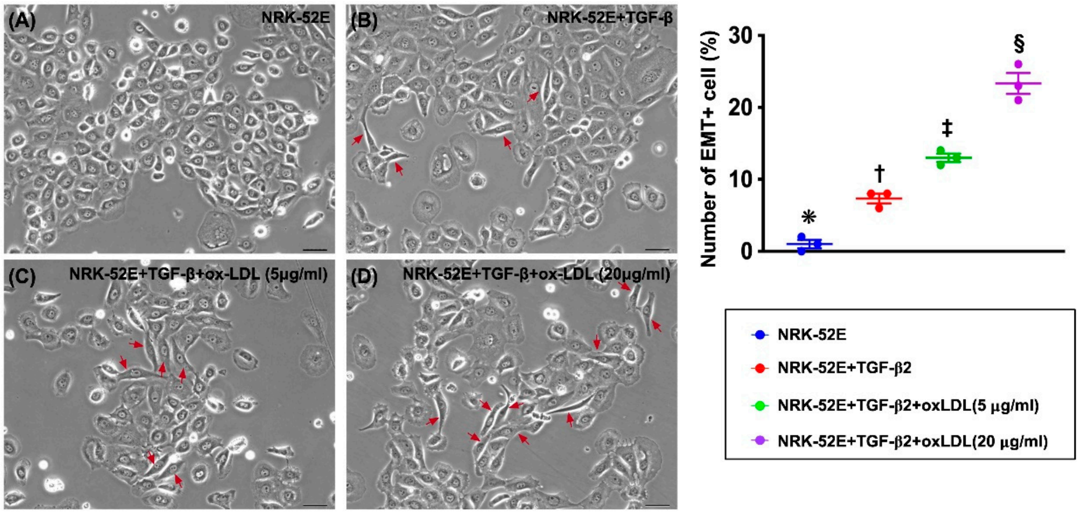


**Figure S2.** Morphological features of NRK-52E cells followed by TGF-β and oxidized LDL stimulation. A to D) Illustrating the optical microscopic finding for identification of morphological changes of NRK-52E cells that adopted from epithelial-like phenotype to spindle-shape phenotype (red arrows) (i.e., indicated mesenchymal-like cells) after 7-day cell culture, especially more prominent in A4 group (red arrows). Right panel) Analytical result of number of morphological spindle shape of mesenchymal-like cells * vs. other groups with different symbols (†, ‡, §), p<0.001. All statistical analyses were performed by one-way ANOVA, followed by Bonferroni multiple comparison post hoc test (n=3 for each group). Symbols (*, †, ‡, §) indicate significance for each other (at 0.05 level).

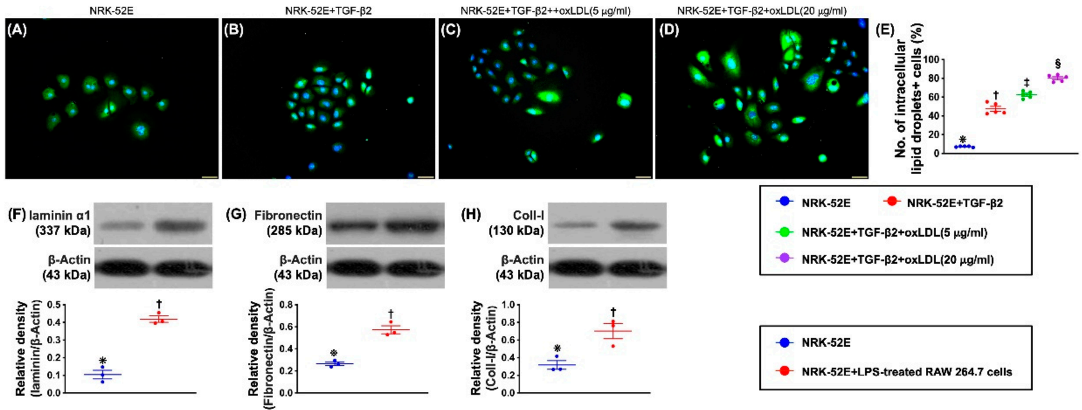


**Figure S3.** Lipid droplets in NRK-52E cells after oxidized LDL treatment and inflammatory cells enhanced ECM production by NRK-52E cells. **(A to D)** Illustrating the immunofluorescent (IF) microscopic finding (400x) for identification of the uptake of oxidized LDL by NRK-52E cells (yellow color). **(E)** Analytical result of number (%) of intracellular lipid droplets, * vs. other groups with different symbols (†, ‡, §), p<0.001. All statistical analyses were

performed by one-way ANOVA, followed by Bonferroni multiple comparison post hoc test (n=5 for each group). Symbols (*, †, ‡, §) indicate significance for each other (at 0.05 level). **(F)** Protein level of laminin, * vs. †, $p<0.001$. **(G)** Protein expression of fibronectin, * vs. †, $p<0.001$. **(H)** Protein expression of collagen type I, * vs. †, $p<0.001$. n = 3 per each group. LPS = lipopolysaccharide; ECM = extracellular matrix.
